# Distribution of controlled unitary quantum gates towards factoring large numbers on today’s small-register devices

**DOI:** 10.1038/s41598-022-25812-z

**Published:** 2022-12-09

**Authors:** Andrei Tănăsescu, David Constantinescu, Pantelimon George Popescu

**Affiliations:** Computer Science and Engineering Department, University Politehnica of Bucharest, Bucharest, 060042 Romania

**Keywords:** Computer science, Quantum information, Qubits

## Abstract

Factoring a 2048-bit number using Shor’s algorithm, when accounting for error correction, reportedly requires 400,000 qubits. However, it is well known that there is yet much time before we will have this many qubits in the same local system. This is why we propose a protocol for distributed quantum computation applicable to small register devices, specifically for the distribution of controlled unitary gates, the key element in the construction of every quantum computation algorithm. We leverage quantum sharing of partial results to obtain a parallel processing scheme, allowing for the first time the quantum distribution of very large gates with thousands of inputs using only small register devices with tens of qubits. In this way, we improve all previous controlled unitary gate distribution approaches, obtaining surprising results. The impact is quantified for recent milestone hardware realizations of quantum processors.

## Introduction

The security of today’s critical communication protocols is generally based upon three pillars: public key encryption, digital signatures and key exchange, the implementation of which is most often based on the difficulty of number theoretic problems such as integer factorization and other hidden subgroup problems^1^. In fact, integer factorization is the computational problem behind today’s most famous cryptosystem, the RSA (Rivest-Shamir-Adleman) cryptosystem, and thus a lot of work has went into developing increasingly sophisticated attacks, based on everything from approximation algorithms to quantum computing. Recently, a PQCrypto 2014 talk^2^ estimated that by 2030 a billion-dollar quantum computer could break 2000-bit RSA in a few hours, a figure that was taken by NIST as *a serious long-term threat to the cryptosystems currently standardized by NIST*^1^, a process which ultimately kicked off a competition to determine the best candidate to replace today’s most popular cryptosystem whose third round finished in late 2021^3^, with a replacement to be ready by 2024. The security of the communication protocols of tomorrow is thus heavily influenced by the advent of quantum computing.

Quantum computing is the branch of computational science that aims to harness quantum phenomena such as superposition and entanglement to perform computational feats, such as the famous polynomial-time Shor factoring algorithm^4^. A recent implementation analysis of Shor’s algorithm^5^ shows that factoring 2048-bit numbers requires at least 400,000 qubits working at least 1 trillion qubit-hours, when error correction is accounted for. While Shor’s algorithm is trivially distributed using sector search, each “thread” still has to have 400,000 qubits, whereas the world’s most powerful quantum processor is reportedly the 127-qubit IBM Eagle r1^6^, itself a far cry from the 5-7 qubit systems freely available in the cloud.

Motivated by similar examples, distributed quantum computation has long studied peer-to-peer coupling in quantum-classical networks using teleportation^7^. One of the first steps in this direction was the distribution of controlled unitary gates^8^, the quantum equivalents of the if programmatic statement. Using the observation that any function can be written as a sequence of if statements, this protocol was recently generalized to allow the distribution at no additional cost of quantum functions^9^, including the Deutsch and Grover oracles, and even the fast modular exponentiation function which is the bottle-necking factor in Shor’s algorithm. While this work shows that the system requirements for distributing a quantum function is the same as for a controlled unitary, the specific implementation of the previously mentioned protocols^8,9^ have the disadvantage that they require a large number of ancillary qubits at the target site.

**Figure 1:**
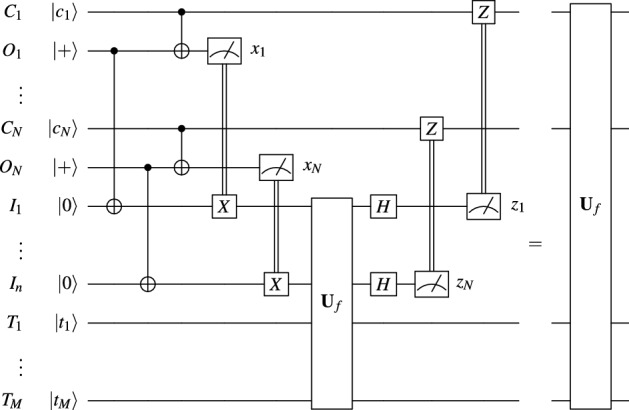
Multipartite distribution of *N*-qubit quantum functions^9^.

The distribution of a quantum function ${\textbf{U}}_f\in {{\,\textrm{End}\,}}\left( {\mathbb {C}}^{2^N}\otimes {\mathbb {C}}^{2^M}\right)$^9^ (and particularly *N*-control unitaries^8^) starts by sharing each of the “control” qubits $C_1,\,\dots ,\,C_N$ with the computer containing the “target” qubits $T_1,\,\dots ,\,T_M$, using *N* additional Bell pairs whose halves we denote by $O_1,\,\dots ,\,O_n$ (at the control site) and $I_1,\,\dots ,\,I_n$ (at the target site). The protocol then locally applies the quantum function ${\textbf{U}}_f$, and finally decommissions the extra qubits, as shown in Fig. 1. This last step is required to avoid residual entanglement, and follows the transfer procedure discussed in^9,10^. Yet, the point of both protocols is not to circumvent register size limits, but rather to allow the processing of non-local data, and as such they both make use of a local copy of the gate. In brief, to distribute a *N*-control Toffoli gate in this way, we still need a $(N+1)$-qubit computer.

**Figure 2:**
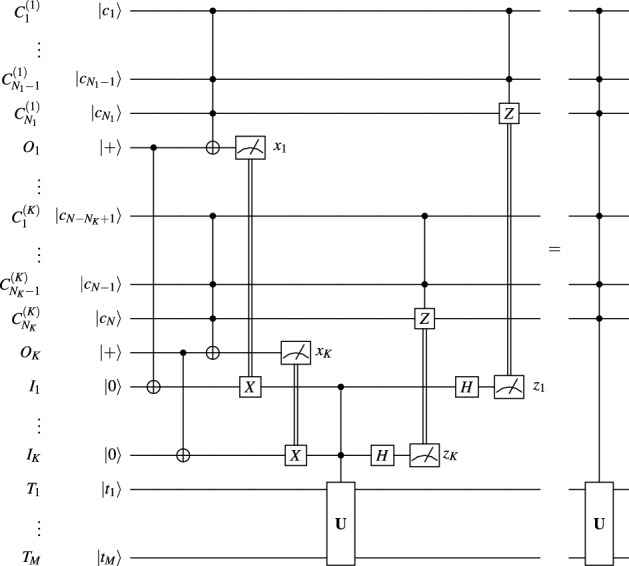
Improved multipartite distribution of *N*-control unitaries^12^.

In the specific case of controlled unitaries that can be further optimized. A clever trick^11^ is to use the relation between the 3-control Toffoli gate and the AND gate to share only partial results of the AND operations. In the bipartite setting, this trick allows expenditure of only 1 Bell pair rather than 2. This idea has been refined by^12^ who eliminated the ancilla and also extended the approach to *N*-control unitary gates. In this setting, the control space is partitioned into *K* groups, ${\mathcal {C}}={\mathcal {C}}_1\otimes \dots \otimes {\mathcal {C}}_K$, each with a possibly different number of qubits $N_i$, ${\mathcal {C}}_i\cong {\mathbb {C}}^{2^{N_i}}$, which we denote $C_1^{(i)},\,\dots ,\,C_{N_i}^{(i)}$ for $1\le i\le K$. Additionally, each control system shares a Bell pair with the target system. In the first step, the protocol applies a local Toffoli gate on each control system targeting the half of the Bell pair. These partial sums are then shared with the target system, where a *K*-control version of the unitary gate is performed. The last step of the protocol is to apply partially classically controlled ${\textbf{Z}}$ gates on the control systems, as depicted in Fig. 2. Yet, the point of this protocol is to optimize for local agglomerations of qubits, so it is only a side effect that it helps our goal. In brief, to distribute a *N*-control Toffoli gate in this way using *n*-qubit control systems we have $K=\frac{N}{n-1}$ and so we still need still need a $(\frac{N}{n-1}+1)$-qubit computer. For example, taking *N*=400,000 and *n*=5, we still need a 100,000-qubit computer. Alternatively, requiring all quantum computers to be of the same size, i.e. setting $n=K+1$, we find $n=\lceil 1+\sqrt{N}\rceil$, i.e. when *N*=400,000 we need 633 computers each having 633 qubits, which is still far from current technology.

In summary, while the literature has optimized distribution of controlled unitaries to some extent, for fully nonlocal controlled unitaries its best solution still comes down to applying the full gate at the target site. Given the lack of any specialized protocol that can work with small register devices, the only recourse is to decompose the *K*-control Toffoli gate into single- and two-qubit gates and then run it through a distributed quantum compiler, such as the one recently developed by IBM researchers^13^ which optimizes the number of remote operations in the compiled circuit using integer programming. While serviceable, this black-box approach offers no particular insight as to how many small-register systems are required to efficiently run a workload requiring full connectivity. Thus, in this paper we set out to provide the first protocols for the distribution of *N*-control Toffoli gates using only small register devices.

## Methods

Throughout this paper we consider a logical quantum system comprised of *N* control qubits, $C_1,\,\dots ,\,C_N$ and *M* target qubits, $T_1,\,\dots ,\,T_M$. The *N* control qubits are split across *K* local physical quantum systems, $S_1,\,\dots ,\,S_K$, while the *M* target qubits are consolidated in a single local physical quantum system, $S_{K+1}$. These $K+1$ local physical systems also include ancillary qubits used for entanglement distribution as well as computation. For convenience, we denote by $N_i$ the number of controls at site $S_i$ for $1\le i\le K$ and relabel them $C_{1}^{(i)},\,\dots ,\,C_{N_i}^{(i)}$. On top of these $K+1$ local physical systems we overlay a communication oriented tree, $G=\left( V,\,E\right)$, where $V=\left\{ S_1,\,\dots ,\,S_{K+1}\right\}$, with the tree rooted at the target site $S_{K+1}$. Without loss of generality, we assume that the nodes are labeled in reverse breadth-first order. For simplicity, we reduce our construction to a single parameter, $1\le B\le K$, corresponding to the branching factor, and all internal nodes will have exactly *B* children, except possibly the smallest indexed one, which may have fewer if the number of nodes is insufficient for them all to have *B* children. As an example, for a balanced binary tree with $K=5$ control systems, the target system has index $S_6$ and communicates with $S_5,\,S_4$, where $S_5$ communicates with $S_3,\,S_2$ and $S_4$ communicates only with $S_1$.

We now use the parametric communication tree to describe a sequence of circuit equivalences. At each step we apply the equivalence in Fig. 2 to a partition of a subset of nodes, based on the subtrees to which they belong. To aid this process, we additionally denote for each $1\le i\le K+1$ the following: let $P_i\subseteq \left\{ 1,\,\dots ,\,K\right\}$ be its children in the tree, let $1\le a_i\le K$ be the smallest index of its children, let $0\le b_i\le B$ be the number of its children, let $1\le p_i\le K+1$ be its parent in the tree, and let ${\hat{P}}_i\subseteq \left\{ 1,\,\dots ,\,K\right\}$ be all the nodes in its subtree (including itself). By the chosen construction and numbering, it follows that $P_i=\left\{ S_{a_i+1},\,\dots ,\,S_{a_i+b_i}\right\}$.

**Figure 3:**
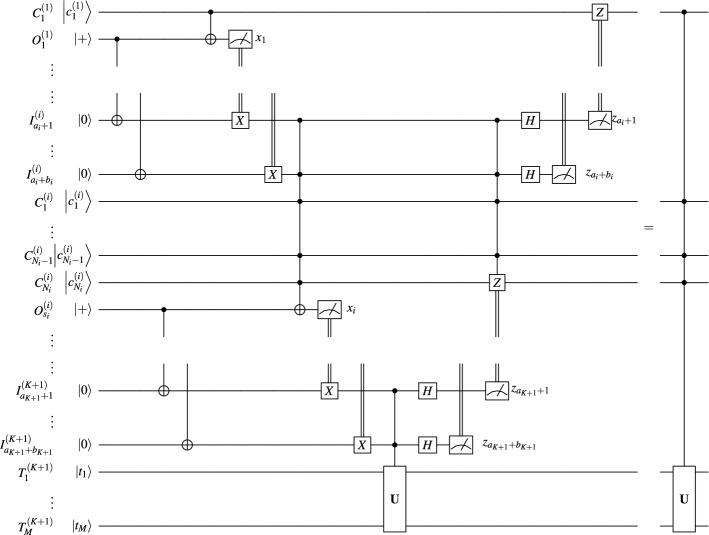
Parallel cascade distribution of controlled unitaries.

### Theorem

The circuit equivalence in Fig. 3 holds.

We provide a proof by induction, following a chain of circuit equivalences aided by Fig. 2, as follows.

### Proof

In the first step we consider the entire controlled unitary gate. Its targets are the qubits $T_1,\,\dots ,\,T_M$ in system $S_{K+1}$, while its controls are the qubits $C_1,\,\dots ,\,C_N$ split across the systems $S_1,\,\dots ,\,S_K$. We partition the controls $C_1,\,\dots ,\,C_N$ into $b_{K+1}$ sets based on the subtree to which their system belongs, i.e. corresponding to the controls in ${\hat{P}}_{a_{K+1}+1},\,\dots ,\,{\hat{P}}_{a_{K+1}+b_{K+1}}$. We then place $b_{K+1}$ Bell pairs, one between each pair of systems $S_{a_{K+1}+j}$ and $S_{K+1}$ for $1\le j\le b_{K+1}$. For each *j*^th^ such Bell pair, we denotte by $O_{a_{K+1}+j}^{(a_{K+1}+j)}$ its half residing within $S_{a_{K+1}+j}$, and by $I_{a_{K+1}+j}^{(K+1)}$ its half residing within $S_{K+1}$. We now apply the equivalence in Fig. 2. This distributes the entire controlled unitary gate targeting $S_{K+1}$, decomposing it into $1\le b_{K+1}\le B$ (possibly non-local) Toffoli gates each targeting one of the $b_{K+1}$ systems $S_{a_{K+1}+j}$.

In the next steps, we perform a similar operation for each nonlocal Toffoli gate that has not yet been distributed. By induction, we assume (and, for the first step, we know) that every such gate corresponds to a subtree rooted at a system $S_i$, i.e. that the involved controls are exactly those belonging to the systems in this subtree, ${\hat{P}}_i$, and that the target is $O_{i}^{(i)}$. We partition these controls into $b_i+1$ sets according to their belonging to ${\hat{P}}_{a_i+1},\,\dots ,\,{\hat{P}}_{a_i+b_i}$ and $S_i$. We then place $b_{i}$ Bell pairs, one between each pair of systems $S_{a_{i}+j}$ and $S_{i}$ for $1\le j\le b_{i}$. For each *j*^th^ such Bell pair, we denotte by $O_{a_{i}+j}^{(a_{i}+j)}$ its half residing within $S_{a_{i}+j}$, and by $I_{a_{i}+j}^{(i)}$ its half residing within $S_{i}$. We now apply the equivalence in Fig. 2, with the first $b_i$ sets acting non-locally, and the local ${\textbf{U}}$ gate acting on the target and local controls in $S_i$. This distributes this nonlocal Toffoli gate controlled by the controls in ${\hat{P}}_i$ and $S_i$ and targeting $O_{i}^{(i)}$, decomposing it into (possibly non-local) $1\le b_{i}\le B$ Toffoli gates, each targeting one of the $b_{i}$ systems $S_{a_{i}+j}$. Notice that this perpetuates the induction assumption, hence at the end of this process all of the gates are local.

Now, at the end of this process, the final circuit equivalence takes the form shown in Fig. 3, completing the proof.$\square$

## Results

The main insight of this paper is that not all *K* controls in a fully non-local *K*-control Toffoli gate need to be brought together. Instead, each site can compute and store the partial sum of one or more subsets of bits. In a chain topology, site *i* receives from site $i-1$ the product of the first $i-1$ bits, factors the *i*^th^ bit into the product, and sends the result forward. This allows us to distribute the *K*-qubit Toffoli gate over a network comprised only of 3-qubit systems, at the cost of execution time numerically equal to *K*. Similarly, in a tree topology with uniform branching factor $B\ge 2$, site *i* receives from subordinate sites $i_1,\,\dots ,\,i_B$ the product of their assigned bits, factors them together with the *i*^th^ bit, and sends the result forward to its superior. This allows us to distribute the *K*-qubit Tofffoli gate over a network comprised only of $(B+2)$-qubit systems, at the cost of execution time $\log _B K$. In particular, when every site has $B+2$ qubits, the execution time is logarithmic: $\log _{B+2} K$, and when the execution time is required to be a fixed constant *t* the qubit count is a fractional power, $K^{1/t}$.
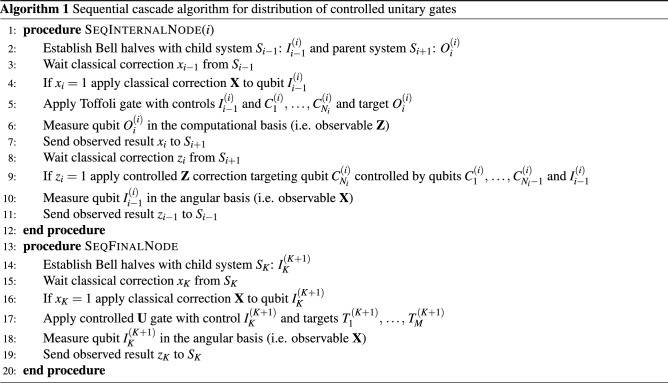


### Cascading Toffoli gates

**Figure 4:**
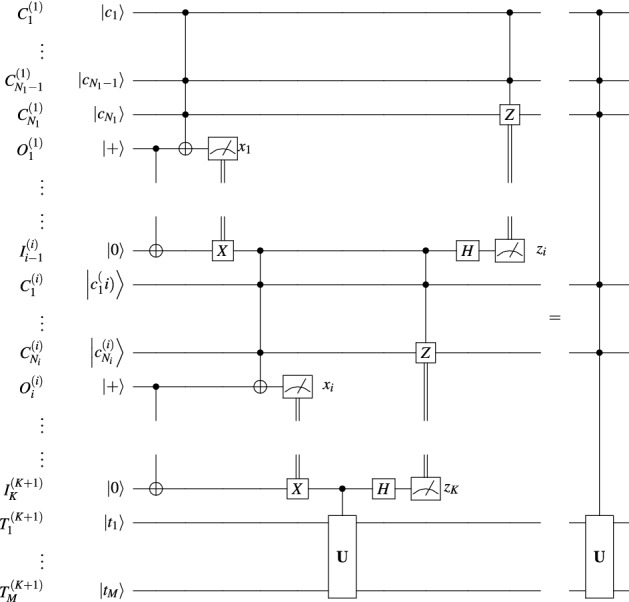
Cascade distribution of controlled unitaries.

The most qubit-efficient rendition of this argument comes in the form of a cascade of Toffoli gates. Based on the equivalence between the statement if($\wedge _{{j=1}^{Nc_{j}}}$==1) then $|\psi \rangle$=${\textbf{U}}|\psi \rangle$; and the cascade statement if($\wedge _{{j=1}^{N_{1}}}c_j$==1) then {... if($\wedge _{j=N-N_{K+1}}^Nc_j$==1) then $|\psi \rangle$=${\textbf{U}}|\psi \rangle$;...}, we note that the unitary gate in the equivalence in Fig. 2 can itself be a controlled unitary. We apply this observation *K* times, each time considering only the controls located at the *i*^th^ site, and targeting not only the target qubits at site $K+1$, but also the qubits in sites $i+1,\,\dots ,\,K$. This produces the equivalence in Fig. 4, a simplified version of Fig. 3 adapted to the path topology.

#### Corollary

The circuit equivalence in Fig. 4 holds.

#### Proof

This immediately follows from the equivalence in Fig. 3 setting $B=1$. $\square$

As can be observed, the *i*^th^ site must have $N_i+2$ qubits for $1<i\le K$, the 1^st^ site must have $N_1+1$ qubits, and the target site must have $M+1$ qubits. In particular, to implement a non-local *N*-control Toffoli gate when all systems have *n* qubits we find $K=\lceil \frac{N-1}{n-2}\rceil$ computers. We can also present this protocol in the form of a distributed algorithm, specifying the actions at each of the local sites $S_i$, as shown in Algorithm 1 where all internal nodes $S_1,\,\dots ,\,S_K$ execute procedure SeqInternalNode and the root $S_{K+1}$ executes SeqFinalNode. Using this protocol we can implement such a gate controlled by *N*=400,000 qubits using 133,333 5-qubit systems, but at a cost of a linear execution time, required by the fact that site $i+1$ necessarily awaits for the input from site *i* before performing its computation. Alternatively, one can use 15,385 28-qubit IBM Falcon r1 systems, etc.

### Parallel cascades

To circumvent the linear execution time, we can set up a different communication tree, $G=\left( V,\,E\right)$, as described in the Methods section, where the vertices correspond to the sites $V=\left\{ 1,\,\dots ,\,K+1\right\}$ with $K+1$ being the root and the directed edges *E* are such that the degree of almost all vertices except the leaves is $B+1$. The resulting circuit is shown in Fig. 3 and the corresponding equivalence is proven in the Methods section.

In this protocol, the time required for the execution of the gate is equal to the depth of the graph. If each site except the sources has an inner degree $B\ge 2$, this depth is $\lceil \log _B K\rceil$. We can also present this protocol in the form of a distributed algorithm, specifying the actions at each of the local sites $S_i$, as shown in Algorithm 2 where all internal nodes $S_1,\,\dots ,\,S_K$ execute procedure ParInternalNode and the root $S_{K+1}$ executes ParFinalNode. In particular, to implement a non-local *N*-control Toffoli gate when all systems have *n* qubits we find $K=\lceil \frac{N+1-B}{n-B-1}\rceil$. Thus, using this protocol we can implement such a gate controlled by $N=400,000$ qubits using 200, 000 5-qubit systems, in execution time $18\tau$ where $\tau$ is the time required for a node’s computation (i.e. the local Toffoli and Hadamard gates and communication with neighbors). Alternatively, one can use 16,667 27-qubit IBM Falcon systems, etc.
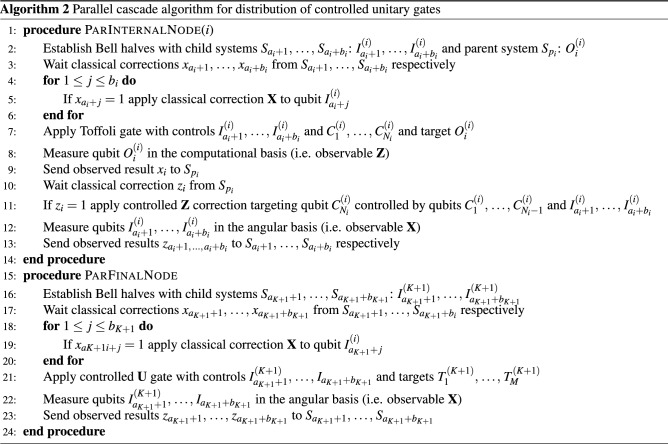


## Discussion

In this subsection we will compare the existing distribution algorithms with the presented one in terms of execution time, number of required maximum local qubits, as well as number of entanglement pairs consumed. Next, we will discuss the applications and implications of the improved distribution algorithm.

We firstly consider the case of a fully non-locally *N*-controlled unitary having *M* target qubits. In this scenario, the protocols of^8,9,12^ reduce to the circuit in Fig. 1. We compare these protocols with the cascade method in Fig. 4 and two variations of the parallel cascade method in Fig. 3: considering a balanced binary tree topology, and a balanced $\sqrt{N}$-tree topology. As it can be seen from Table 1, our protocols greatly reduce the requirement on local subsystem dimension, either to its square root while maintaining constant time, or even to a constant but at the cost of logarithmic time.

**Table 1:** Comparison of distribution protocols for a fully non-locally *N*-controlled unitary.

	Ancillary Qubits	Maximum Qubits Locally	EPR pairs consumed	Time
Existing Algorithms^8,9,12^ (Fig. 1)	2*N*	$N+M$	*N*	${\mathcal {O}}\left( 1\right)$
Sequential Cascade (Fig. 4)	2*N*	$\max \left\{ 3,\,M+1\right\}$	*N*	${\mathcal {O}}\left( N\right)$
Binary Parallel Cascades (Fig. 3)	2*N*	$\max \left\{ 4,\,M+2\right\}$	*N*	${\mathcal {O}}\left( \log N\right)$
$\sqrt{N}$ Parallel Cascades (Fig. 3)	2*N*	$\lceil \sqrt{N}+M\rceil$	*N*	${\mathcal {O}}\left( 1\right)$

Now, we consider the case of a non-local *N*-controlled unitary where *N* controls are spread across *K* local subsystems and all *M* target qubits are consolidated into one subsystem. We denote the largest number of controls assigned to either of these subsystems as *n*. In this scenario, the protocols of^8,9^ no longer coincide with that of^12^. We again compare the existing protocols with the cascade method in Fig. 4 and the same two variations of the parallel cascade method in Fig. 3. As it can be seen from Table 2, our protocols once again greatly reduce the requirement on local subsystem dimension even beyond^12^, either to its square root while maintaining constant time, or even to a constant but at the cost of logarithmic time.

**Figure 5:**
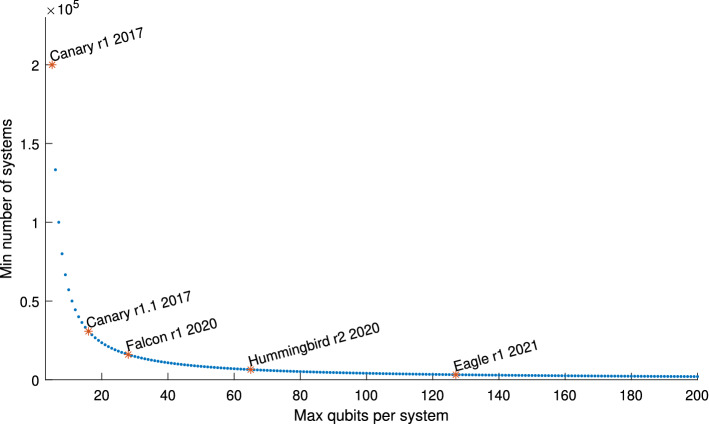
Minimal number of required systems to distribute a 400,000-controlled unitary using Algorithm 2 as required to factor a 2048-bit number using Shor’s algorithm, emphasizing the timeline of IBM quantum processors^14^.

We visually represent the number of systems required to distribute a 400,000-controlled unitary gate using Algorithm 2 in Fig. 5, emphasizing the timeline of recent milestone hardware realization of quantum processors. For example, we can see that we would need a network of 200,000 5-qubit Canary r1 processors (or other equivalents, as commonly available on the market) or, equivalently instead, a network of 3,226 127-qubit IBM Eagle r1 processors.

**Table 2:** Comparison of distribution protocols for a fully non-local *N*-controlled unitary.

	Ancillary Qubits	Maximum Qubits Locally	EPR pairs consumed	Time
Full Clustering^8,9^ (Fig. 1)	2*N*	$N+M$	*N*	${\mathcal {O}}\left( 1\right)$
Local Clustering^12^ (Fig. 2)	2*K*	$\max \left\{ n+1,\,K+M\right\}$	*K*	${\mathcal {O}}\left( 1\right)$
Sequential Cascade (Fig. 4)	2*K*	$\max \left\{ n+2,\,M+1\right\}$	*K*	${\mathcal {O}}\left( K\right)$
Binary Parallel Cascades (Fig. 3)	2*K*	$\max \left\{ n+3,\,M+2\right\}$	*K*	${\mathcal {O}}\left( \log K\right)$
$\sqrt{N}$ Parallel Cascades (Fig. 3)	2*K*	$\max \left\{ \lceil n+\sqrt{K}\rceil ,\,\lceil M+\sqrt{K}\rceil \right\}$	*K*	${\mathcal {O}}\left( 1\right)$

In conclusion, the protocols presented in this paper compare favorably to the state of the art, greatly reducing restrictions on local system dimension. Consequently, if all quantum operations in Shor’s algorithm such as modular exponentiation were to be decomposed as products of controlled unitaries, with a logarithmic (18x) increase in execution time, we could run Shor’s algorithm to factor 2048-bit numbers using 200,000 5-qubit systems. This highlights the impact of future work related to the distribution of not just controlled unitaries, but actual quantum functions such as fast modular exponentiation which could lead to the experimental implementations of Shor’s algorithm distributed across a network of small registry devices.

For future work we propose the analysis of the noise introduced by entanglement distribution across a network with tens of thousands of quantum computers, for example considering computation fidelity when using only diluted EPR states. In fact, it is not clear how this compares to how noise scales in large-scale quantum computers with hundreds of thousands of qubits.

## Data Availability

The datasets generated during the current study will be made available from the corresponding author on reasonable request.
